# Assessment of high- and low-risk histopathologic subtypes of basal cell carcinoma using artificial intelligence and dermatologist evaluation

**DOI:** 10.1016/j.jdin.2026.03.014

**Published:** 2026-04-15

**Authors:** Victor Liang, Parasto Shahrouki, Daniel Nejad, Eva Backman, Magdalena Claeson, Åsa Ingvar, Isabelle Krakowski, Kari Nielsen, Jenna Pakka, John Paoli, Niki Radros, Mari Salmivuori, Sam Polesie

**Affiliations:** a Department of Dermatology and Venereology, Institute of Clinical Sciences, Sahlgrenska Academy, University of Gothenburg, Gothenburg, Sweden; b Department of Dermatology and Venereology, Sahlgrenska University Hospital, Gothenburg, Region Västra Götaland, Sweden; c Division of Dermatology, Department of Clinical Sciences, and Lund University Cancer Center, LUCC, Faculty of Medicine, Lund University, Lund, Sweden; d Department of Dermatology, Skåne University Hospital, Lund, Sweden; e Department of Medicine Solna, Karolinska Institutet, Stockholm, Sweden; f Department of Dermatology, Theme Inflammation, Karolinska University Hospital, Stockholm, Sweden; g Theme Cancer, Karolinska University Hospital, Stockholm, Sweden; h Department of Dermatology, Allergology and Venereology, Helsinki University Hospital and University of Helsinki, Helsinki, Finland; i Center for Digital Health, Sahlgrenska University Hospital, Gothenburg, Region Västra Götaland, Sweden

**Keywords:** artificial intelligence, basal cell carcinoma, convolutional neural network, machine learning

## Abstract

**Background:**

Basal cell carcinoma (BCC) is the most common skin cancer, and accurate histopathologic risk stratification is essential for treatment selection. Convolutional neural networks (CNNs) demonstrate promise in image analysis, yet few studies have evaluated classification of BCC subtypes.

**Objective:**

To assess the diagnostic performance of a CNN for binary classification of high-*versus* low-risk histopathologic subtypes of BCC and compare it with dermatologists.

**Methods:**

In this retrospective single-center study, 1580 dermoscopic images of histopathologically confirmed BCCs were used to train a preconditioned CNN. A test set (*n* = 249) was evaluated by 9 dermatologists. Performance was assessed using area under the receiver operating characteristic curve (AUC ROC). Logistic regression assessed associations between predefined tumor features and histopathologic subtype.

**Results:**

The CNN achieved an AUC of 0.75 (95% confidence interval [CI], 0.69-0.81) comparable to the dermatologists (AUC 0.79, 95% CI: 0.73-0.84, *P* = .18). Bumpy surface topography, clinical and dermoscopic ulceration and ill-defined border as well as dermoscopic focused vessels and white porcelain areas were associated with high-risk BCC, whereas dermoscopic unfocused vessels, erosions, and pigmentation were associated with low-risk BCC.

**Limitations:**

Retrospective single-center design and limited metadata for the CNN.

**Conclusion:**

CNNs appear useful for binary classification of BCC histopathologic subtypes.

## Graphical abstract

**Figure:**
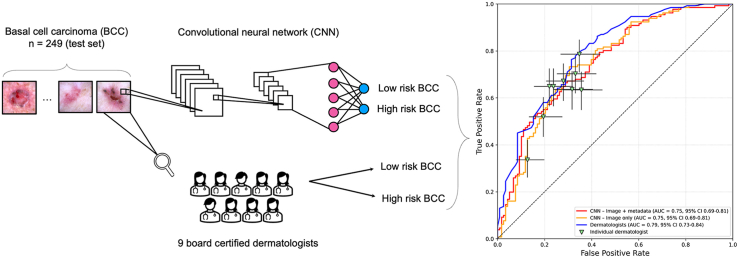

**Capsule Summary**

• Convolutional neural networks show potential to assist histopathologic risk stratification in basal cell carcinoma, building on earlier dermatologic image analysis research.
• This study suggests neural networks may help classify high- and low-risk basal cell carcinoma subtypes, offering a potential adjunct for diagnostic triage and guiding biopsy decisions.

## Introduction

Basal cell carcinoma (BCC) is the most common malignancy and the increasing incidence presents a significant public health challenge and imposes considerable economic strain on healthcare systems.^1^^,^^2^ BCC is often diagnosed through clinical assessment, but the use of dermoscopy increases diagnostic accuracy compared to the naked eye examination and specific dermoscopic features have been associated to different histopapathologic subtypes.^3^, ^4^, ^5^

The general consensus is that high-risk histopathological subtypes of BCC (HR-BCC) are managed with surgical excision including Mohs micrographic surgery (MMS) to ensure complete clearance. On the other hand, in many healthcare systems, low-risk histopathological subtypes of BCC (LR-BCC) can also be managed using less costly and readily available nonsurgical treatment modalities, including curettage and electrodessication as well as cryosurgery.^6^^,^^7^

The gold standard for histopathologic subtype classification prior to the therapeutical management currently relies on skin biopsy which requires follow-up visits, increases cost, and prolongs the time to definitive treatment. In increasingly resource-strained healthcare systems, there is a growing need for individualized treatment selection and more resource-efficient management strategies requiring a more readily available, less invasive and accurate diagnostic technique. In many European healthcare systems, including Sweden, preoperative punch biopsies for BCCs are not routinely performed. Instead, these tumors are generally managed based on their clinical and dermoscopic appearance, and the specimen is then sent for histopathologic confirmation. However, in cases where there is a high suspicion of a more aggressive tumor BCC subtype, particularly in the head and neck region, a preoperative punch biopsy is recommended. This is especially important because such tumors may be eligible for MMS.^8^ This highlights the need for preoperative tools to help clinicians determine the histopathologic aggressiveness of these tumors. Consequently, an accurate pre-treatment estimation of the histopathologic BCC subtype is important for correct and cost-effective management.^6^

AI (artificial intelligence) models have demonstrated non-inferior, and in some cases superior, performance to clinicians for image-based skin cancer diagnostics with convolutional neural networks (CNNs) being the most widely applied machine learning (ML) modality.^9^^,^^10^ However, most studies have focused on binary classification of melanoma *versus* benign lesions or on broader multi-class lesion classification tasks. To date, only a few niche, task-specific CNN applications have been investigated, including models designed to estimate preoperative melanoma thickness or assess preoperative differentiation in cutaneous squamous cell carcinoma (cSCC).^11^^,^^12^ Importantly, existing studies using clinical or dermoscopic images for BCC diagnostics have primarily evaluated BCC as a single category within multi-class classification frameworks, rather than distinguishing between BCC histopathologic subtypes.^13^, ^14^, ^15^ The primary objective of this study was to evaluate the potential of a CNN in differentiating between HR-BCC and LR-BCC. By comparing its discriminative performance to that of dermatologists, we seek to ascertain whether this represents a viable application for further algorithmic refinement and future clinical implementation.

## Material and methods

This retrospective observational study included data from the Pathology Department at Sahlgrenska University Hospital, consisting of histopathologically verified BCC tumors diagnosed between 2016 and 2022. Patient-level data collected included histopathologic subtype, patient age and anatomic tumor location. In Sweden, histopathologic subtyping of BCC is routinely performed, and all lesions included were evaluated by dermatopathologists (Supplementary Appendix S1, available via Mendeley at https://data.mendeley.com/datasets/n8bfdwkhy6/1). In summary, HR-BCC included micronodular, infiltrative, morpheaform, and basosquamous variants, whereas LR-BCC included nodular and superficial variants.

Dermoscopic and clinical images were retrieved from the hospital imaging archive and linked to the corresponding pathology reports. Dermoscopic images were primarily acquired using DermLite DL3 and DL4 devices (3 Gen Inc) and primarily the iPhone 8 Plus smartphone (Apple Inc). All images underwent manual quality review to verify diagnostic adequacy. Images of insufficient quality, incorrectly labeled tumors, colliding tumors, duplicates, previously biopsied prior to image or non-polarized imaging were excluded. Cases lacking valid images or a specified histopathologic subtype were also excluded.

A CNN (Supplementary Appendix S2, available via Mendeley at https://data.mendeley.com/datasets/n8bfdwkhy6/1) was developed using Python (version 3.10, Python Software Foundation) and the PyTorch deep learning framework. A pretrained ResNet-50 architecture was employed as the backbone for transfer learning. Only dermoscopic images were included for model development. The dataset was divided into a combined training and validation set and an independent test set through randomization by Python Random Module. Images with excessive black background were automatically trimmed using custom Python code. To leverage pretrained ImageNet weights and enable stable optimization, images were initially resized close to the native input resolution of ResNet-50 starting from 254 × 254 progressively increasing to 448 × 448 pixels during training. Data augmentation was adapted from Bo *et al.*^16^ In addition to the image-only model, a metadata-augmented model was trained using the same cross-validation procedure as for the image-only model, incorporating the anatomic location of each lesion as a structured input feature. The metadata were encoded through an embedding layer and fused with the image feature representation before the final classification layer.

The test set was reviewed independently by 9 board-certified dermatologists in the time interval from September to December 2025. All invited dermatologists had received training in subtyping BCC during residency and/or dermoscopy courses, providing a relevant clinical benchmark for this task.

Each case included a dermoscopic image, a clinical close-up image, and annotations for anatomic location, allowing for assessments under clinically relevant conditions (Supplementary Appendix S3, available via Mendeley at https://data.mendeley.com/datasets/n8bfdwkhy6/1). All images were presented to the readers at their original pixel resolution. Readers classified each lesion as HR-BCC or LR-BCC and rated their diagnostic confidence on a five-level ordinal scale (*very uncertain, moderately uncertain, average, moderately certain and very certain*). In addition, they assessed a set of prespecified clinical and dermoscopic features using standardized categorical response options. The tumor characteristic *ill-defined border* was assessed based on either the clinical image, dermoscopic image, or both, depending on which image provided the clearest margin visualization. Features of *any blue pigment* and *vessels within ulceration* were only accessible for assessment if *pigment* and *dermoscopic ulceration* were considered present, respectively. Readers were required to complete all items, with no option to skip variables. To ensure consistency in interpretation, dermatologists were provided with an instructional manual detailing all predefined tumor features (Supplementary Appendix S4, available via Mendeley at https://data.mendeley.com/datasets/n8bfdwkhy6/1).

Descriptive statistics were used to summarize patient demographics, anatomic tumor locations, and the distribution of LR-BCC and HR-BCC across the datasets. Model *versus* reader performance was evaluated using the area under the receiver operating characteristic curve (AUC). For all ROC analyses, HR-BCC served as the positive class. AUC ROC values were calculated for (1) the CNN model using dermoscopic images only, (2) the metadata-augmented CNN model incorporating anatomic location in addition to dermoscopic images, and (3) combined dermatologist group. Dermatologist performance was analyzed both individually and as a combined group. A certainty-weighted performance metric for the binary histopathologic risk classification task was constructed by applying numerical weights to the five-level diagnostic confidence scale (Supplementary Appendix S5, available via Mendeley at https://data.mendeley.com/datasets/n8bfdwkhy6/1).

Statistical differences between AUC ROC values were assessed using DeLong’s test, comparing the image-only CNN with the combined dermatologist group and the metadata-augmented CNN with the combined dermatologist group. To assess associations between predefined clinical and dermoscopic features and histopathologic risk category, univariable logistic regression analyses were performed with HR-BCC defined as the positive outcome, estimating odds ratios (ORs) with corresponding 95% confidence intervals (CIs). Interobserver variability among dermatologists in the evaluation of predefined clinical and dermoscopic features was assessed using Fleiss kappa (κ). The strength of agreement was interpreted according to conventional benchmarks: poor (κ ≤ 0), slight (κ > 0-0.20), fair (κ > 0.20-0.40), moderate (κ > 0.40-0.60), substantial (κ > 0.60-0.80), and almost perfect (κ > 0.80).^17^^,^^18^

For each tumor characteristic, dermatologist consensus was defined as the category receiving the highest number of votes among the 9 readers, provided that at least 2 dermatologists selected that category. If no category received ≥2 votes or a tie occurred, the feature was coded as missing. The same definition was used for both the baseline characteristics and the logistic regression analyses. All analyses were performed using R statistical software (version 4.4.3, R Foundation for Statistical Computing).

## Results

**Fig 1:**
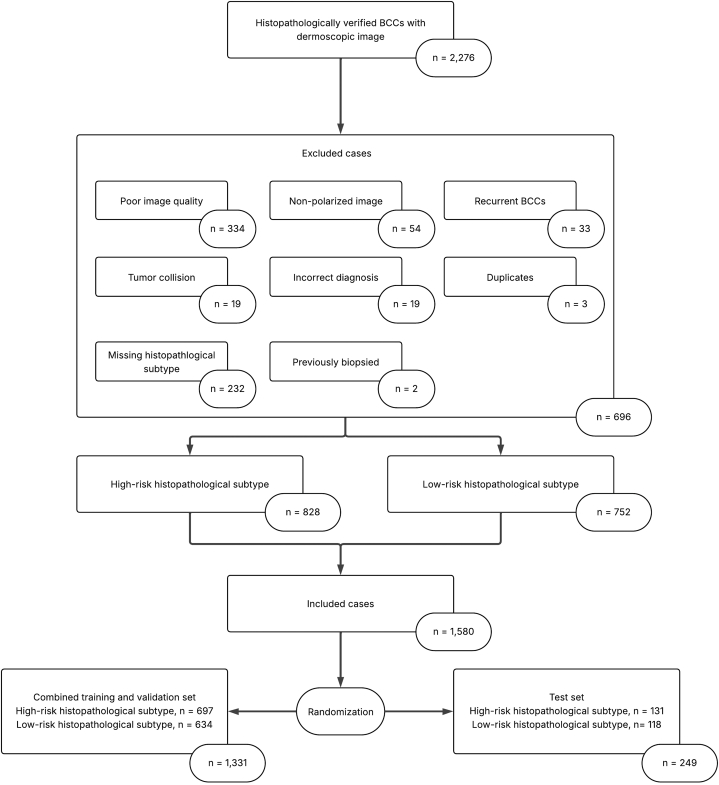
Flowchart showing inclusion and exclusion of BCC tumors. *BCC*, Basal cell carcinoma.

Nine independent board-certified dermatologists participated as readers, representing different dermatology departments. The readers experience within dermatology ranged from 4 to 25 years (median 20 years). Following application of the exclusion criteria, a total of 1580 unique BCC tumors met the inclusion criteria, of which 828 were HR-BCC (52.4%) and 752 were LR-BCC (47.6%). A test set of 249 lesions (131 HR-BCC and 118 LR-BCC) (Supplementary Appendix S6, available via Mendeley at https://data.mendeley.com/datasets/n8bfdwkhy6/1) was obtained through random assignment, with the remaining lesions allocated to the combined training and validation set which included 697 HR-BCC and 634 LR-BCC. Randomization was performed by pre-specifying the number of tumors to be placed in the test set and assigning all remaining cases to the combined dataset (Fig 1).

The median age at BCC diagnosis was 80 years in both groups. There were 141 (56.6%) males in the test set and 758 (56.9%) males in the combined training and validation set. The face and neck region was the most common anatomic location for BCC in both groups, accounting for 138 lesions (55.4%) in the test set and 697 lesions (50.3%) in the combined training and validation set. This was followed by the trunk with 37 lesions (14.9%) and 275 lesions (20.7%) in the respective sets.

When combining dermatologists’ assessments of the predefined clinical and dermoscopic characteristics, flat surface was the most prevalent topographic feature, while shiny white structures was the most frequently observed dermoscopic finding (Table I).

**Table I:** General tumor characteristics

General characteristics	Test set (*N* = 249)	Training and validation set (*N* = 1331)
Median age, y (IQR)	80.0 (75.0-86.0)	80.0 (75.0-85.0)
Male sex—no. (%)	141 (56.6)	758 (56.9)
High-risk histopathological subtype—no. (%)	131 (52.6)	697 (52.4)
Low-risk histopathological subtype—no. (%)	118 (47.4)	634 (47.6)
Anatomic location—no. (%)		
Ears	17 (6.8)	61 (4.6)
Periocular	0 (0.0)	2 (0.2)
Face and neck	138 (55.4)	670 (50.3)
Feet and toes	0 (0.0)	2 (0.2)
Hands and fingers	1 (0.4)	4 (0.3)
Lips	1 (0.4)	4 (0.3)
Lower extremities	31 (12.4)	164 (12.3)
Scalp	5 (2.0)	45 (3.4)
Trunk	37 (14.9)	275 (20.7)
Upper extremities	19 (7.6)	104 (7.8)
Tumor characteristics—present features∗		
Clinical features—no. (%)		
Surface topography		
Flat	108 (43.4)	
Raised	85 (34.1)	
Depressed	21 (8.4)	
Bumpy	26 (10.4)	
Crust	75 (30.1)	
Ulceration	67 (26.9)	
Ill-defined border†	111 (44.6)	
Dermoscopic features—no. (%)		
Focused vessels	114 (45.8)	
Unfocused vessels	80 (32.1)	
Ulceration	94 (37.8)	
Vessels within ulceration	57 (22.9)	
Erosions	44 (17.7)	
Pigmented structures	73 (29.3)	
Any blue pigment	39 (15.7)	
Shiny white structures	185 (74.3)	
White porcelain area	35 (14.1)	

∗ Present defined as category with the highest number of votes and ≥2 dermatologists selected that category. If no category received ≥2 votes or a tie occurred, the feature was coded as missing (no consensus).

† Ill-defined border could be assessed using clinical and/or dermoscopic images.

**Fig 2:**
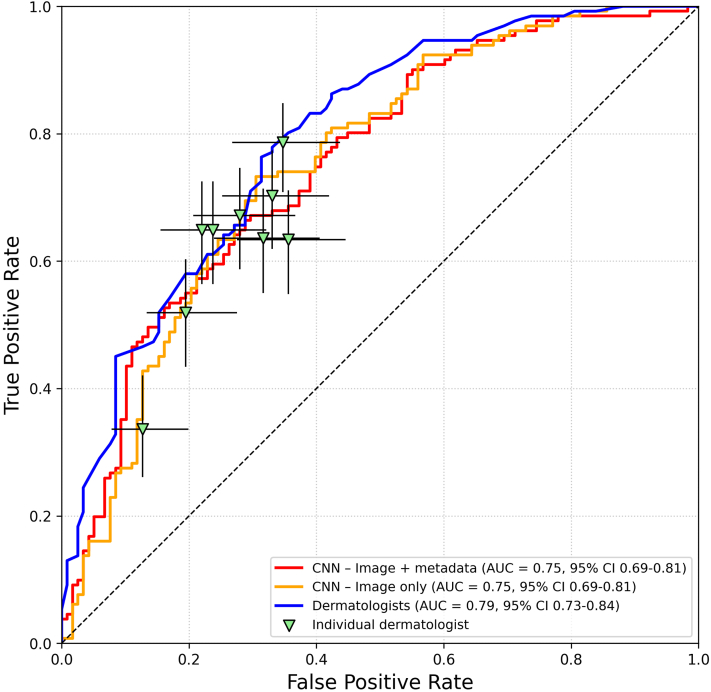
AUC-ROC curves for CNN models and dermatologists. True positive refers to correctly classifying high-risk histopathological basal cell carcinoma. *Red curve* represents CNN model based on dermoscopic image with known anatomic location; *yellow line* represents CNN model trained on dermoscopic images only; *blue line* represents the combined dermatologist group, adjusted for certainty level; *green triangles* represent individual dermatologist performance with corresponding 95% confidence interval. *AUC ROC*, Area under the receiver operating characteristic curve; *CI*, confidence interval; *CNN*, convolutional neural network.

For all ROC analyses, the true positive rate corresponded to the proportion of HR-BCC cases correctly classified, with LR-BCC served as the negative class. The CNN trained on dermoscopic images alone achieved an AUC of 0.75 (95% CI: 0.69-0.81). Adding anatomic location information did not change performance, with the combined model also yielding an AUC of 0.75 (95% CI: 0.69-0.81). The combined dermatologist group AUC was 0.79 (95% CI: 0.73-0.84) which did not significantly differ from any of the CNN models (*P* = .18 and *P* = .18, respectively) (Fig 2).

**Fig 3:**
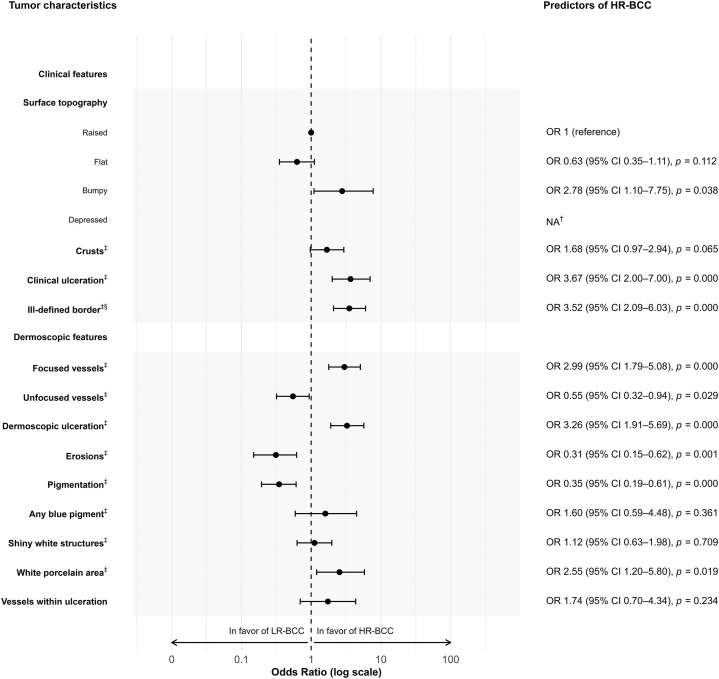
Univariate logistic regression analysis displayed as a forest plot with predictors of HR-BCC. ^†^Complete separation occurred for the category “Depressed,” with no cases classified as low-risk (ie, all 21 lesions with a depressed surface were of high-risk subtype). ^‡^Variables had “Absent” as reference category. Odds ration values represent “Present” features. ^§^Ill-defined border could be assessed using clinical and/or dermoscopic images. *HR-BCC*, High-risk histopathological subtypes of BCC; *LR-BCC*, Low-risk histopathological subtypes of BCC; *OR*, odds ratio.

All ORs were derived from univariate logistic regression analyses, using *raised* as the reference category for surface topography and *absent* as the reference category for all other features. Among the surface topography categories, *bumpy* was the only feature associated with HR-BCC, OR 2.78 (95% CI: 1.10-7.75, *P* = .038). Notably, the *depressed* surface topography exhibited complete separation (21 HR-BCC, 0 LR-BCC). Other statistically significant features associated with HR-BCC included *clinical ulceration* (OR: 3.67, 95% CI: 2.00-7.00, *P* < .001), *ill-defined border* (OR: 3.52, 95% CI: 2.09-6.03, *P* < .001), *focused vessels* (OR: 2.99, 95% CI: 1.79-5.08, *P* < .001), *dermoscopic ulceration* (OR” 3.26, 95% CI: 1.91-5.69, *P* < .001), and *white porcelain area* (OR: 2.55, 95% CI: 1.20-5.80, *P* = .019). Statistically significant features associated with LR-BCC were exclusively dermoscopic features, including *unfocused vessels* (OR: 0.55, 95% CI: 0.32-0.94, *P* = .029), *erosions* (OR: 0.31, 95% CI: 0.15-0.62, *P* = .001), and *pigmentation* (OR: 0.35, 95% CI: 0.19-0.61, *P* < .001) (Fig 3).

*Clinical and dermoscopic ulceration*, and *pigmentation* exhibited substantial agreement, with clinical ulceration (κ = 0.68, 95% CI: 0.66-0.70), and pigmentation (κ = 0.64, 95% CI: 0.62-0.66) showing the highest κ values among clinical and dermoscopic features, respectively. All remaining variables showed moderate or fair agreement. *Unfocused vessels* had the lowest interobserver agreement (κ = 0.22, 95% CI: 0.19-0.24) (Table II).

**Table II:** Interobserver agreement among dermatologists assessed using Fleiss kappa

Tumor characteristics	Fleiss kappa(κ)
Clinical features	
Surface topography	0.41 (95% CI: 0.40-0.42)
Crusts	0.56 (95% CI: 0.54-0.58)
Clinical ulceration	0.68 (95% CI: 0.66-0.70)
Ill-defined border∗	0.49 (95% CI: 0.47-0.51)
Dermoscopic features	
Focused vessels	0.57 (95% CI: 0.55-0.59)
Unfocused vessels	0.22 (95% CI: 0.19-0.24)
Dermoscopic ulceration	0.61 (95% CI: 0.59-0.63)
Vessels within ulceration	0.33 (95% CI: 0.28-0.38)
Erosions	0.45 (95% CI: 0.43-0.48)
Pigmentation	0.64 (95% CI: 0.62-0.66)
Any blue pigment	0.46 (95% CI: 0.41-0.51)
Shiny white structures	0.36 (95% CI: 0.33-0.38)
White porcelain area	0.30 (95% CI: 0.28-0.32)

Agreement categories were defined as follows: poor (κ ≤ 0), slight (κ > 0-0.20), fair (κ > 0.20-0.40), moderate (κ > 0.40-0.60), substantial (κ > 0.60-0.80), and almost perfect (κ > 0.80).

∗ Ill-defined border could be assessed using clinical and/or dermoscopic images.

## Discussion

This single-center, retrospective study provides new insights into the use of pretrained CNN for binary classification of histopathological BCC subtypes, demonstrating diagnostic performance that performed on par with an experienced group of board-certified dermatologists. With the rising incidence of BCC and the growing need for individualized, resource-efficient care, non-invasive and readily accessible methods with reliable diagnostic accuracy for BCC risk stratification are increasingly important. In parallel, the expanding use of teledermoscopy and digital triage pathways represents a potentially suitable application area for niche, task-specific CNN models such as the ones presented in this study.^19^

Additional metadata with anatomic location in the CNN model did not improve AUC. This is in contrast to a study published by Pacheco *et al*, who reported improved diagnostic performance when clinical metadata were added to image-based models. However, their study included multiple clinical data variables, such as anatomic location, age, pruritus as well as bleeding of lesion.^20^ The absence of performance gain from anatomic location metadata suggests that this variable provides limited discriminatory value for separating HR- and LR-BCC.

Several clinical and dermoscopic features including *bumpy surface topography*, *ill-defined border*, *clinical* and *dermoscopic ulceration*, *focused vessels* and *white porcelain areas* were associated with high-risk subtypes, whereas the dermoscopic features of *unfocused vessels*, *erosions*, and *pigmentation* were associated with low-risk lesions. Features such as bumpy tumor topography have recently been described as a predictor of facial HR-BCC, while an ill-defined border has been described as a feature of both facial HR-BCC and LR-BCC.^21^^,^^22^ White porcelain area, sometimes also referred to as hypopigmented structureless areas, have in prior studies been associated to HR-BCC.^23^ In line with previous studies, leaf-like pigmentation and erosions are more commonly observed in LR-BCC.^24^

Key strengths of this study include the use of a histopathologically confirmed ground truth, independent assessments by multiple dermatologists, and a focused evaluation of task-specific, clinically relevant diagnostic dilemmas that reflect real-world decision-making challenges. This study also has noteworthy limitations. First, its retrospective, single-center design may limit generalizability, as image acquisition protocols and dermoscopy devices may differ across dermatology clinics. Second, only dermoscopic images were used for CNN model training, whereas clinicians had access to both clinical and dermoscopic images; the comparison therefore reflects different information inputs. However, providing additional clinical images alongside dermoscopy does not necessarily improve CNN performance. A possible explanation is that clinical photographs are less standardized than dermoscopic images and vary considerably in angle, distance, and background room illumination.^25^^,^^26^ Third, although the dermatologists’ assessments were performed independently, interobserver variability was moderate for many features, which may affect the reliability of feature-based associations. Fourth, patients included in this investigation were primarily of Nordic origin, comprising mostly individuals with Fitzpatrick skin types 1-3, which may limit generalizability to populations with darker skin types. However, BCC occurs predominantly in individuals within this population, which partially mitigates this limitation.^27^ Fifth, histopathologic stratification followed a Swedish tradition of classifying BCCs.^28^ Pathology assessment may vary between different healthcare systems, but the general distinction between high- and low-risk subtypes does align with international classifications.^29^ Finally, in Sweden, a considerable amount of LR-BCC, especially superficial BCC, are frequently managed based on clinical and dermoscopic assessment alone without subsequent histopathological confirmation. As this dataset was derived from a pathology registry, these cases are underrepresented, potentially introducing selection bias toward lesions deemed clinically difficult to subtype or aggressive enough to warrant biopsy. Conversely, this subset may represent a promising target for the future development of augmented intelligence, as these BCC tumors could potentially benefit from decision-support solutions.

## Conclusion

The present investigation demonstrates that CNN-based discrimination of HR-BCC and LR-BCC using dermoscopic images is feasible, with performance comparable to that of experienced dermatologists While not intended for immediate clinical implementation, these findings suggest that AI-based risk stratification could complement preoperative clinical assessment in selected settings, particularly in healthcare systems where subtype differentiation guides management. We consider this work a focused, hypothesis-generating step that provides a foundation for future prospective, multicenter studies evaluating the clinical utility and integration of such approaches.

## Conflicts of interest

None disclosed.

## References

[1] Wang R., Chen Y., Shao X. (2025). Burden of skin cancer in older adults from 1990 to 2021 and modelled projection to 2050. JAMA Dermatol.

[2] Wu X., Elkin E.E., Marghoob A.A. (2015). Burden of basal cell carcinoma in USA. Future Oncol.

[3] Reiter O., Mimouni I., Gdalevich M. (2019). The diagnostic accuracy of dermoscopy for basal cell carcinoma: a systematic review and meta-analysis. J Am Acad Dermatol.

[4] Reiter O., Mimouni I., Dusza S., Halpern A.C., Leshem Y.A., Marghoob A.A. (2021). Dermoscopic features of basal cell carcinoma and its subtypes: a systematic review. J Am Acad Dermatol.

[5] Ahnlide I., Zalaudek I., Nilsson F., Bjellerup M., Nielsen K. (2016). Preoperative prediction of histopathological outcome in basal cell carcinoma: flat surface and multiple small erosions predict superficial basal cell carcinoma in lighter skin types. Br J Dermatol.

[6] National Comprehensive Cancer Network (NCCN NCCN clinical practice guidelines in oncology: basal cell skin cancer. Version 1.2026. https://www.nccn.org/professionals/physician_gls/pdf/nmsc.pdf.

[7] Paoli J., Gyllencreutz J.D., Fougelberg J. (2019). Nonsurgical options for the treatment of basal cell carcinoma. Dermatol Pract Concept.

[8] Kappelin J., Nielsen K., Nilsson F., Bjellerup M., Ahnlide I. (2020). Surgical treatment of basal cell carcinoma: a case series on factors influencing the risk of an incomplete primary excision. J Eur Acad Dermatol Venereol.

[9] Salinas M.P., Sepúlveda J., Hidalgo L. (2024). A systematic review and meta-analysis of artificial intelligence versus clinicians for skin cancer diagnosis. NPJ Digit Med.

[10] Esteva A., Kuprel B., Novoa R.A. (2017). Dermatologist-level classification of skin cancer with deep neural networks. Nature.

[11] Polesie S., Gillstedt M., Ahlgren G. (2021). Discrimination between invasive and in situ melanomas using clinical close-up images and a De Novo Convolutional Neural Network. Front Med (Lausanne).

[12] Liang V., Engström E., Gillstedt M. (2025). Assessing differentiation in cutaneous squamous cell carcinoma: a machine learning approach. JAAD Int.

[13] Widaatalla Y., Wolswijk T., Adan F. (2023). The application of artificial intelligence in the detection of basal cell carcinoma: a systematic review. J Eur Acad Dermatol Venereol.

[14] Naeem A., Anees T., Fiza M., Naqvi R.A., Lee S.W. (2022). SCDNet: a deep learning-based framework for the multiclassification of skin cancer using dermoscopy images. Sensors (Basel).

[15] Iqbal I., Younus M., Walayat K., Kakar M.U., Ma J. (2021). Automated multi-class classification of skin lesions through deep convolutional neural network with dermoscopic images. Comput Med Imaging Graph.

[16] Ha Q., Liu B., Liu F. (Published online October 2020). Identifying melanoma images using EfficientNet ensemble: winning solution to the SIIM-ISIC melanoma classification challenge. arXiv e-prints.

[17] Landis J.R., Koch G.G. (1977). The measurement of observer agreement for categorical data. Biometrics.

[18] Fleiss J.L. (1971). Measuring nominal scale agreement among many raters. Psychol Bull.

[19] Foltz E.A., Ludzik J., Leachman S. (2024). Revolutionizing skin cancer triage: the role of patient-initiated teledermoscopy in remote diagnosis. Cancers (Basel).

[20] Pacheco A.G.C., Krohling R.A. (2020). The impact of patient clinical information on automated skin cancer detection. Comput Biol Med.

[21] Camela E., Ilut Anca P., Lallas K. (2023). Dermoscopic clues of histopathologically aggressive basal cell carcinoma subtypes. Medicina (Kaunas).

[22] Ceder H., Backman E., Marghoob A. (2024). Importance of both clinical and dermoscopic findings in predicting high-risk histopathological subtype in facial basal cell carcinomas. Dermatol Pract Concept.

[23] Popadić M. (2015). Dermoscopy of aggressive basal cell carcinomas. Indian J Dermatol Venereol Leprol.

[24] Longo C., Lallas A., Kyrgidis A. (2014). Classifying distinct basal cell carcinoma subtype by means of dermatoscopy and reflectance confocal microscopy. J Am Acad Dermatol.

[25] Gillstedt M., Mannius L., Paoli J. (2022). Evaluation of melanoma thickness with clinical close-up and dermoscopic images using a convolutional neural network. Acta Derm Venereol.

[26] Rios-Duarte J.A., Diaz-Valencia A.C., Combariza G., Feles M., Peña-Silva R.A. (2024). Comprehensive analysis of clinical images contributions for melanoma classification using convolutional neural networks. Skin Res Technol.

[27] Naik P.P., Desai M.B. (2022). Basal cell carcinoma: a narrative review on contemporary diagnosis and management. Oncol Ther.

[28] Jernbeck J., Glaumann B., Glas J.E. (1988). [Basal cell carcinoma. Clinical evaluation of the histological grading of aggressive types of cancer]. Lakartidningen.

[29] Cameron M.C., Lee E., Hibler B.P. (2019). Basal cell carcinoma: epidemiology; pathophysiology; clinical and histological subtypes; and disease associations. J Am Acad Dermatol.

